# Supplementary material for: The turbulent future brings a breath of fresh air
Source: Nat Commun. 2023 Jun 22;14:3735. doi: 10.1038/s41467-023-39298-4 (PMC10287702; doi:10.1038/s41467-023-39298-4)
Supplement: Supplementary file 1 — Supplementary information [file 41467_2023_39298_MOESM1_ESM.pdf]

SUPPLEMENTARY MATERIAL

The turbulent future – a breath of fresh air

Camilla W. Stjern*[1], Øivind Hodnebrog[1], Gunnar Myhre[1], Ignacio Pisso[2]

[1] CICERO Center for International Climate Research, Oslo, Norway

[2] Norwegian Institute for Air Research (NILU), Kjeller, Norway

*Corresponding author: Camilla W. Stjern; camilla.stjern@cicero.oslo.no

Supplementary materials include:

Tables S1 and S2

Figures S1 – S6

Experiment name	Details
**BASE**	Present-day (year 2000) conditions and emissions
**CO2x2**	CO2-concentrations doubled compared to year 2000 values (i.e. CO2 level at 720 ppm)
**BCx10**	Tenfold increase in BC emissions compared to year 2000.
**SO4x5**	Fivefold increase in SO2 and SO4 emissions compared to year 2000.

Table S1: Simulations conducted with CESM2-CAM6 in this study. For each experiment, we performed one simulation with a set-up fully coupled with an ocean model (cpl), as well as one simulation with fixed sea-surface temperatures (fsst). The fsst simulations were run for 15 years, for which the first 5 were considered spin-up and not included in the analyses. For the cpl simulations we first ran for 60 years to stabilize the model, then each simulation was continued for 70 years, for which the last 20 were used in the analyses.

Experiments	Models included	Total no. of models
**historical**	'CanESM5-CanOE', 'CanESM5', 'CESM2-FV2', 'CESM2', 'CESM2-WACCM-FV2', 'CESM2-WACCM', 'EC-Earth3-AerChem', 'GFDL-ESM4', 'GISS-E2-1-G', 'GISS-E2-1-H', 'GISS-E2-2-H', 'HadGEM3-GC31-LL', 'HadGEM3-GC31-MM', 'INM-CM4-8', 'INM-CM5-0', 'IPSL-CM6A-LR-INCA', 'MIROC-ES2H', 'MIROC-ES2L', 'MPI-ESM-1-2-HAM', 'NorESM2-LM', 'NorESM2-MM', 'TaiESM1', 'UKESM1-0-LL'	23
**SSP119**	'CanESM5', 'GFDL-ESM4', 'GISS-E2-1-G', 'GISS-E2-1-H', 'MIROC-ES2L'	5
**SSP245**	'CanESM5-CanOE', 'CanESM5', 'GFDL-ESM4', 'GISS-E2-1-G-CC', 'GISS-E2-1-G', 'GISS-E2-1-H', 'INM-CM4-8', 'INM-CM5-0', 'MIROC-ES2L', 'NorESM2-LM', 'NorESM2-MM', 'TaiESM1', 'UKESM1-0-LL'	13
**SSP370**	'CanESM5-CanOE', 'CanESM5', 'CESM2', 'CESM2-WACCM', 'EC-Earth3-AerChem', 'GFDL-ESM4', 'GISS-E2-1-G', 'GISS-E2-1-H', 'INM-CM4-8', 'INM-CM5-0', 'MIROC-ES2L', 'MPI-ESM-1-2-HAM', 'NorESM2-LM', 'NorESM2-MM', 'TaiESM1', 'UKESM1-0-LL'	16
**SSP585**	'CanESM5-CanOE', 'CanESM5', 'GFDL-ESM4', 'GISS-E2-1-G', 'GISS-E2-1-H', 'INM-CM4-8', 'INM-CM5-0', 'MIROC-ES2L', 'NorESM2-LM', 'NorESM2-MM', 'TaiESM1', 'UKESM1-0-LL'	12

Table S2: Models included in the analyses of PBL height changes in CMIP6 data. The CMIP6 variable used is named “bldep”. Trend analyses are based on monthly mean output.

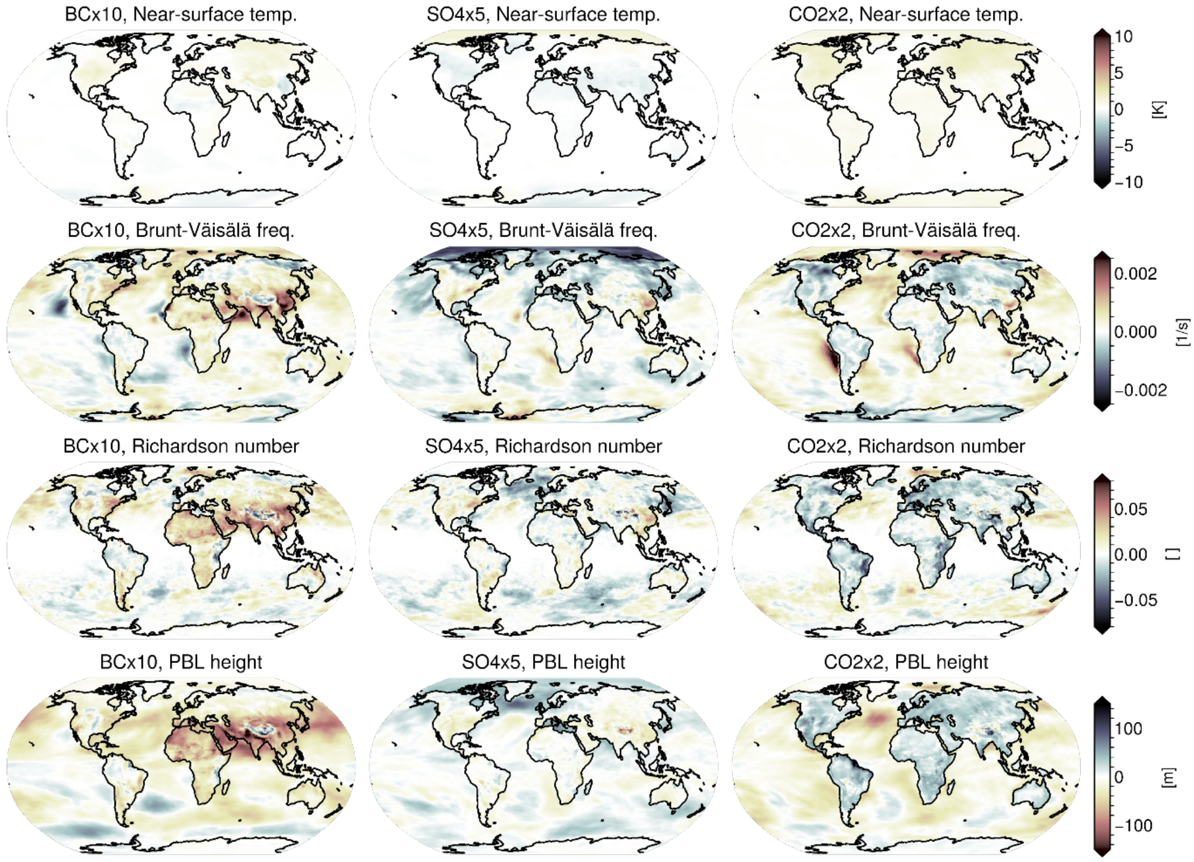

Figure S1: Global changes for simulations with fixed sea surface temperatures. Changes (columns correspond to BCx10, CO2x2 and SO4x5, respectively) in near-surface temperature, Brunt-Väisälä frequency N (indicating lower-atmospheric stability) at the 936 hPa level, Richardson number Ri (the ratio of turbulence-suppressing static stability to turbulence-generating vertical shear) at the 936 hPa level and planetary boundary layer (PBL) height.

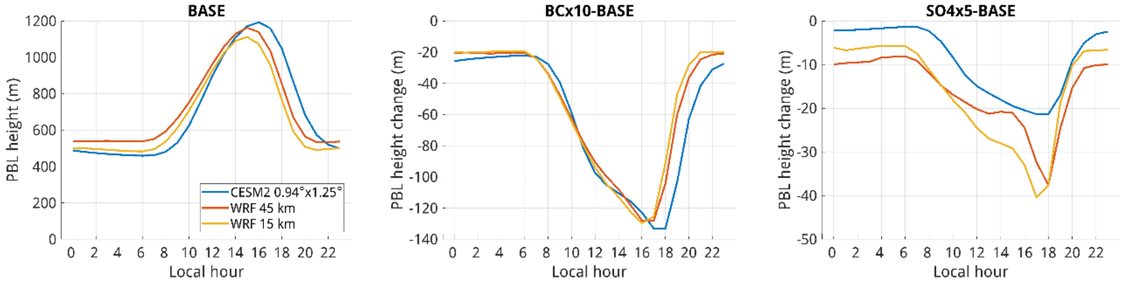

Figure S2: Diurnal evolution of baseline PBL height (left) and change in PBL height for perturbations of BC (middle) and SO_4 (right). Results are averaged over the fine (15 km) resolution domain in WRF (region shown in Fig. S3), which is approximately same as the East China region, for fixed-SST simulations of the CESM2-CAM6 model on which results in the main manuscript are based, as well as for WRF simulations with horizontal resolutions of 45 km and 15 km, respectively. In comparison CESM2-CAM6 has a resolution of around 100 km at these latitudes. All simulations are averaged over years 5-9. Note different scales.

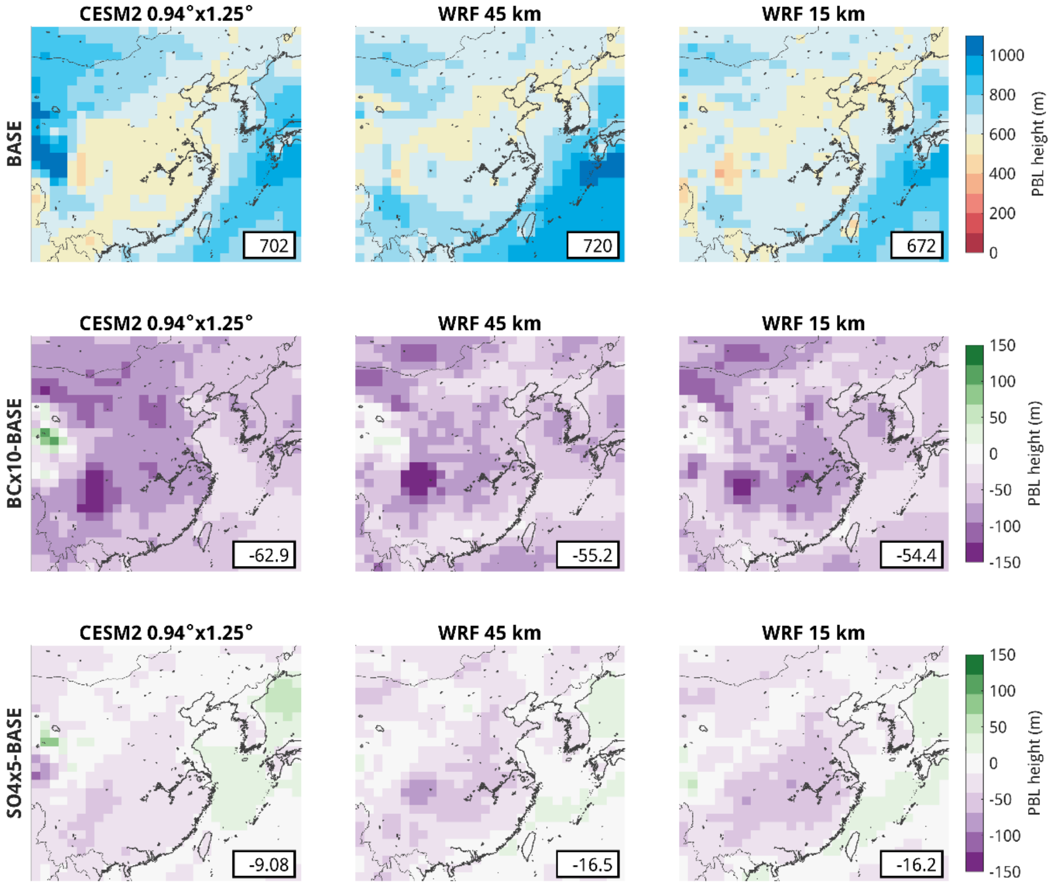

Figure S3: Geographical distribution in baseline PBL height (top row) and change in PBL height for perturbations of BC (middle row) and SO_4 (bottom row). WRF results are regridded to the resolution of CESM2, and boxes give the mean value over the region shown, which is the extent of the 15 km WRF domain and approximately covers the East China region. All simulations are averaged over years 5-9 in fixed-SST configuration.

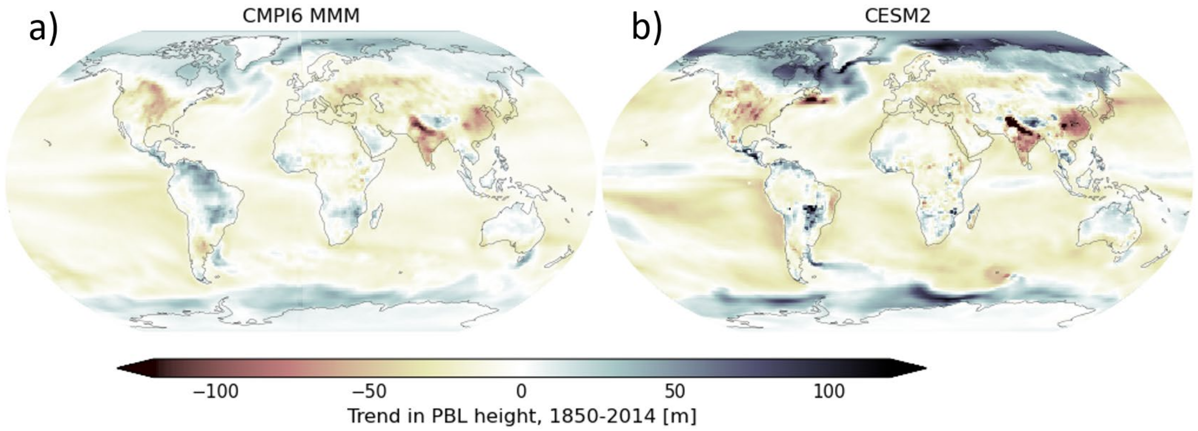

Figure S4: Historical PBLH changes. Change in boundary layer height over the historical period (linear trend from 1850 to 2014) in a) the CMIP6 ensemble (average of models as listed in Table S2) and in b) CESM2-CAM6.

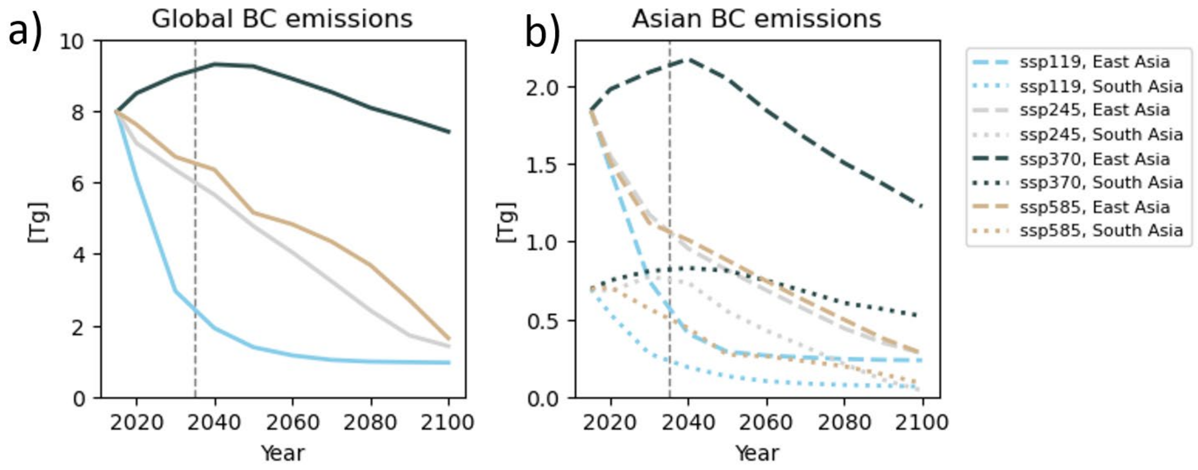

Figure S5: Future BC concentration changes. Global and Asian emissions of BC in all available SSPs.

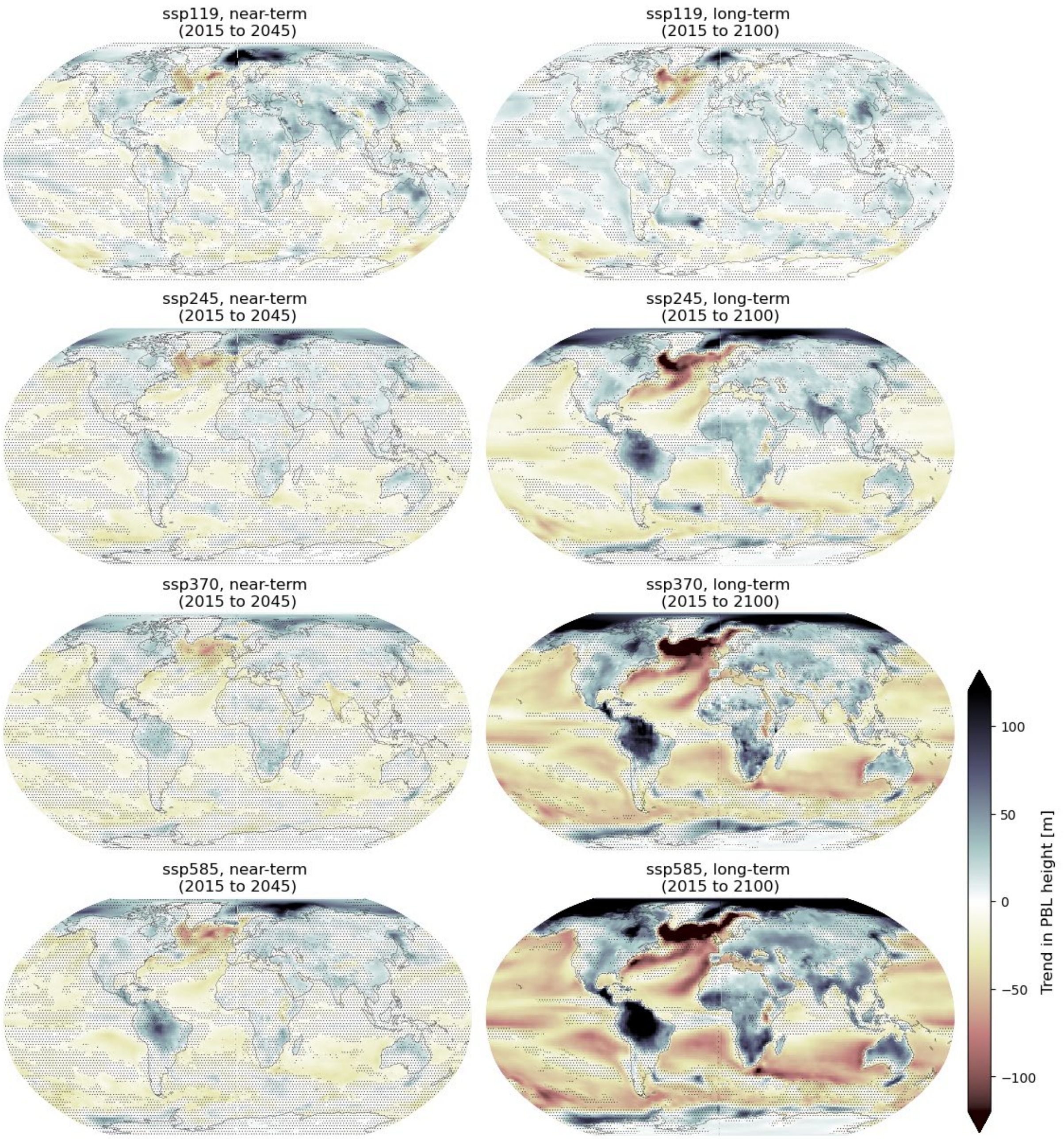

Figure S6: Future PBL height changes. Near-term (2015 to 2045) and long-term (2015-2100) trends in boundary layer height in all available SSPs. Hatching where less than 75% of the models agree on the sign of the change
